# Total Body Capacitance for Estimating Human Basal Metabolic Rate in an Egyptian Population

**Published:** 2016-03

**Authors:** Samir M. Abdel-Mageed, Ehab I. Mohamed

**Affiliations:** 1 Department of Physics, Faculty of Science, Alexandria University, Egypt;; 2 Department of Medical Biophysics, Medical Research Institute, Alexandria University, Egypt

**Keywords:** Total body capacitance, basal metabolic rate, bioelectric impedance analysis, calorimetry

## Abstract

Determining basal metabolic rate (BMR) is important for estimating total energy needs in the human being yet, concerns have been raised regarding the suitability of sex-specific equations based on age and weight for its calculation on an individual or population basis. It has been shown that body cell mass (BCM) is the body compartment responsible for BMR. The objectives of this study were to investigate the relationship between total body capacitance (TBC), which is considered as an expression for BCM, and BMR and to develop a formula for calculating BMR in comparison with widely used equations. Fifty healthy nonsmoking male volunteers [mean age (± SD): 24.93 ± 4.15 year and body mass index (BMI): 25.63 ± 3.59 kg/m^2^] and an equal number of healthy nonsmoking females matched for age and BMI were recruited for the study. TBC and BMR were measured for all participants using octopolar bioelectric impedance analysis and indirect calorimetry techniques, respectively. A significant regressing equation based on the covariates: sex, weight, and TBC for estimating BMR was derived (R=0.96, SEE=48.59 kcal, and *P*<0.0001), which will be useful for nutritional and health status assessment for both individuals and populations.

## INTRODUCTION

The principle of measuring the electric properties of the whole body and its segments was introduced by Barnet (1), who first detected the association between changes of the hydration status and changes of total body resistance (R) and capacitive reactance (X_c_). The X_c_ of biological tissues is known to be the opposition to the instantaneous flow of electric current caused by total body capacitance (TBC), which can be considered as an expression for body cell mass (BCM). Nyboer *et al.* (2) found relations between changes of bioelectrical impedance (Z), which is the vector sum of R and X_c_, and dynamic changes of pulsatile blood flow, arterial pulse waveforms, and respiration. They then applied the principals of bioelectric impedance analysis (BIA), which denotes the measurement of changes of Z across limbs, organs, and other body sites to detect changes of dynamic blood volume, to the study of body composition using static total body Z measurements (3). To date, there is no evidence in the scientific literature on a usage of the association between TBC and BCM to scrutinize changes of basal metabolic rate (BMR) on individual or population basis.

Determining BMR is important for estimating total energy needs in the human being. The widely used sex-specific equations for calculating BMR are based on the anthropometric parameters: age and weight (4-7). However, several studies have raised concerns regarding the suitability of these equations to calculate BMR on an individual basis in a specific age group (8, 9). A criticism for this was that the measured values of weight alone do not account for the variability in the metabolic active mass (i.e., BCM) (10), which has been found to be the best single predictor of BMR (11, 12). The objectives of the present study were to investigate the relationship between TBC and BMR for healthy men and women and, based on this relation, to produce a formula for calculating BMR. Estimations of BMR using this equation will be compared with estimations produced using WHO/FAO/UNU equations (5).

## SUBJECTS AND METHODS

### Study Population

The study population consisted of 100 healthy nonsmoking Egyptian volunteers (50 men and 50 women) with an age range between 19 and 37 year living in the city of Alexandria, Egypt. Volunteers were recruited randomly from among persons participating in various health programs carried out at the Department of Medical Biophysics, Medical Research Institute, Alexandria University, Alexandria – Egypt. Before the study, they were informed individually about the nature and purpose of the experimental procedure and informed written consent was obtained from each participant. The Ethics Committee of the Medical Research Institute, Alexandria University approved the study protocol.

### Anthropometric Measurements

For all participants, body weight (kg) (participants clothed in underwear, bare feet) was measured using a digital scale that was sensitive to the nearest 0.01 kg (Electronic Body Scale, TCS–200–RT, China). Height (m) was measured using a stadiometer. The body mass index (BMI) was expressed as weight/height^2^ (kg/m^2^). Circumferences of waist (cm) and hips (cm) were measured using a tape measure, and the waist-to-hip ratio was calculated. Skinfold thickness was measured using a Holtain caliper (Bryberian, UK) and the sum of four skinfolds (mm) was calculated (i.e., biceps, triceps, subscapular, and supra-iliac skinfolds).

### Octopolar Bioelectric Impedance Analysis (OBIA)

**Figure 1:**
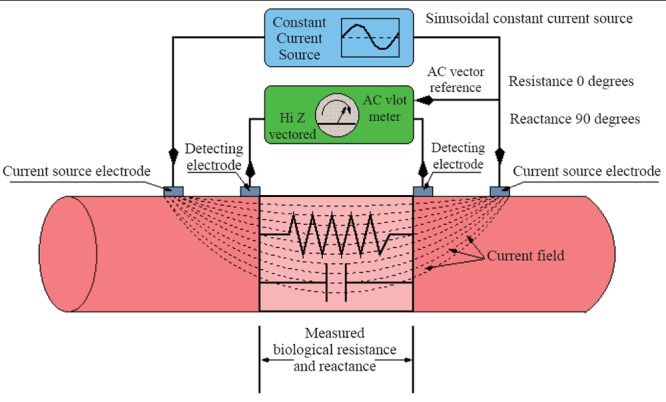
Schematic representation of the bioelectric impedance analysis (BIA) measuring technique, where a sinusoidal constant current (~800 μA at 50 kHz) is passed between the outer two electrodes, while the biological resistance (R) and reactance (X_c_) are measured between the inner electrodes. Total body capacitance (TBC) is measured on basis of the assumption that soft tissues behave as simple RC parallel circuit.

Body-composition measurements were carried out using an Octopolar Bioelectric Impedance Analyzer (InBody 720, Biospace Co., Ltd., Seoul, Korea), which is an eight-point tactile electrode system that separately measures impedance of the arms, trunk, and legs at six different frequencies (1, 5, 50, 250, 500, and 1000 kHz). The basic principles of bioelectric impedance analysis (BIA) technique are shown by the classical schematic representation in Figure 1.

Before measurement, participants wiped the bottom of their feet with an electrolyte tissue. Then, they were instructed to stand, lightly dressed, on the scale while holding the handrails with metal grip electrodes, thereby providing contact with a total of eight electrodes (two for each foot and hand). The participants fully extended their arms at an abduction angle of approximately 20 degrees laterally for less than two minutes (13). The instrument showed R, X_c_, and TBC measurements on the paper report of each participant.

### Indirect Calorimetry

**Figure 2:**
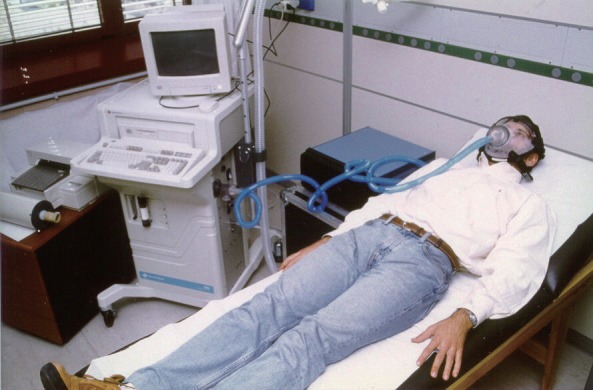
Experimental arrangement for measuring basal metabolic rate (BMR) using indirect calorimetry technique. Rates of oxygen consumption (VO_2_) and carbon dioxide (VCO_2_) production are determined using computerized metabolic chart.

The Horizon metabolic chart (MMC, SensorMedics Inc., Anaheim, CA, USA) was used for determining oxygen consumption (VO_2_) and carbon dioxide production (VCO_2_) as illustrated in Figure 2. Subjects were introduced to the equipment and given a briefing on the experimental protocol before the day of the measurement. They were advised to abstain from coffee and other nicotine containing food or beverage, heavy meals, and strenuous exercise in the evening prior to measurement. They were informed to undergo 12 hours overnight fast and to reach the study center without undue exertion. Subjects were then allowed to lie down quietly and relaxed for 30 minutes before measurement commenced. Female subjects were measured within the first ten days of menstrual cycle (the first day of menstruation taken as day 1). All measurements were carried out between 6.00 and 8.30 am in an air conditioned room with temperatures and humidity ranging between 23 – 26°C and 758 – 770 mmHg, respectively. BMR was calculated using the formula of Weir (14), which is given by:

[Eq. 1] $$\text{BMR}\;\text{(kcal/min)}=3.941\times {\text{VO}}_{2}\;\text{(l/min)}+1.106\times {\text{VCO}}_{2}\;\text{(l/min)}$$

### Data Analysis

Statistical analyses were performed using the StatView^®^ statistical package (SAS Institute Inc., Cary, NC, USA). Measurements were expressed as mean ± SD. Unpaired student’s *t*-test of significance was used to compare differences between male and female participants for anthropometric, metabolic, and electric variables. Differences were considered to be significant if *P*<0.05.

Bivariate linear regression analysis was performed to examine the interrelations among variables using simple and partial correlation coefficients (R). The associations between BMR and sex, weight, and TBC were modeled using multiple linear regression analysis. The regression coefficient (β), standard error of estimation (SEE), and significance level (P) were determined for independent variables added simultaneously. Significance was accepted at *P*<0.05 for single terms.

A sex-specific equation for BMR based on three independent variables (i.e., sex, weight, and TBC) was developed and its cumulative correlation coefficient (R) and standard error of estimation (SEE) were calculated. Sex was included as a variable in the equation, which was coded as 1 for men and 2 for women, to avoid developing separate equations of BMR for males and for females, as we described earlier (15). Scattergrams and regression plots for BMR as a function of TBC were produced for male and female participants of average weight. For comparing estimations of BMR using the developed equation with estimations produced using the widely used WHO/FAO/UNU equations at appropriate age ranges (5), scattergrams and regression plots for BMR as a function of weight were also produced for male and female participants of average TBC.

## RESULTS AND DISCUSSION

The anthropometric, metabolic, and electric characteristics for male and female participants are shown in Table 1. Males were significantly heavier and taller than females (*P*<0.0001). Males also had a significantly higher waist-to-hip ratio than females, which was due to a significantly higher waist circumference (87.72 ± 11.35 mm for males versus 80.79 ± 11.33 mm for females; *P*<0.01) but not hip circumference (101.11 ± 6.42 mm for males versus 103.94 ± 10.25 mm for females; NS). Males had lower fat content in comparison with females as shown by a lower significant sum of four skinfolds.

**Table 1:** Anthropometric, metabolic, and electric characteristics of all study participants

	Males (n=50)	Females (n=50)

**Age** (yr)	24.93 ± 4.15	27.40 ± 5.28
**Weight** (kg)	81.06 ± 13.18^a^	68.94 ± 12.63
**Height** (m)	1.78 ± 0.07^a^	1.63 ± 0.05
**Body Mass Index** (BMI, kg/m^2^)	25.63 ± 3.59	26.14 ± 4.96
**Waist-to-Hip**	0.87 ± 0.07^a^	0.78 ± 0.06
**Sum of Four Skinfolds** (mm)	42.54 ± 22.22^a^	63.70 ± 26.08
**Oxygen Consumption** (VO_2_, ml/min)	271.36 ± 24.78^a^	219.76 ± 26.28
**Carbon Dioxide Production** (VCO_2_, ml/min)	216.71 ± 24.04^a^	172.44 ± 19.92
**Basal Metabolic Rate** (BMR, kcal/day)	1309.10 ± 118.96^a^	1056.78 ± 122.50
**Total Body Resistance** (R, Ω)	458.55 ± 38.75^a^	570.12 ± 53.75
**Total Body Reactance** (X_c_, Ω)	56.88 ± 5.65^a^	59.14 ± 5.39
**Total Body Impedance** (Z, Ω)	462.09 ± 38.88^a^	573.21 ± 54.02
**Total Body Capacitance** (TBC, pF)	856.98 ± 111.04^a^	577.74 ± 84.85

All values are expressed as mean ± SD.

a *P*<0.0001 versus female group

Both VO_2_ and VCO_2_, and consequently BMR, were significantly higher for males than for females (*P*<0.0001). The electric parameters: R, X_c_, and Z but not TBC, which was significantly higher, were significantly lower for males than for females (*P*<0.0001). The differences in these metabolic and electric properties between both groups mainly derive from the stature and metabolic activity, especially muscle mass, for men compared to women.

Estimating the energy expenditure for an individual or for a population is important because it is a major determinant of food energy requirements. Since BMR constitutes about 60% to 70% of the total energy expenditure, it has been widely used as the basis of a factorial method for deriving energy requirements of any given population. Determining BMR have gained attention since the publication of the WHO/FAO/UNU expert consultation report (5), which adopted the principle relying on estimates of energy expenditure rather than energy intake for calculating energy requirements for adults.

The BMR of an individual is known as the minimum metabolic activity required for maintaining life, whether the individual is sleeping, resting or working. BMR is measured under standardized resting conditions: bodily and mentally at rest, 12-14 hours after a meal, and in a neutral thermal environment. However, in practice it is far more difficult to achieve the conditions of ‘basal metabolism’ than it is to define them (16).

In practice, BMR is not commonly measured instead, sex-specific equations based on age and weight, are used for its calculation (4-7, 17). In the largest and most comprehensive analysis of BMR to date, Schofield *et al.* (18) reviewed some 11,000 BMR measurements in the literature and developed equations for males and females, which were later adopted for use in the WHO/FAO/UNU report (5). While the Schofield equations estimate BMR accurately for many individuals in mild climate, they are said to be less accurate for estimating BMR for populations especially those living in tropical areas. Most calculated values were derived from North Americans and Europeans and their analysis revealed an overestimation for BMR of about 10% in Asian Indians (19-21). This overestimation may mainly derive from either racial differences or because of the fact that body weight alone does not account for the metabolically active mass BCM (8, 10).

An initial multiple linear regression model was used to determine the effect of simultaneously adding the covariates sex, weight, TBC, and age on BMR as the dependent variable. Table 2 shows that sex, weight, and TBC but not age, which did not attain statistical significance, were significantly associated with BMR. The sex was used in the analysis so that the results of BMR can be presented in a single regression model, rather than separated by sex. The final regression model included only the covariates: sex, weight, and TBC (R = 0.96, SEE = 48.59 kcal, and P < 0.0001), thus our proposed equation developed for calculating BMR is given by:

[Eq. 2] BMR (kcal)=705.760-101.923×Sex+5.554×Weight (kg)+0.298×TBC (pF)

**Table 2:** Coefficients of the initial multiple linear regression for predictors of basal metabolic rate (BMR) added simultaneously

	β	SEE	*P* Value

**Intercept**	731.143	134.538	*<0.0001*
**Sex** (Male/Female)	-95.393	35.632	*0.0090*
**Weight** (kg)	5.688	0.797	*<0.0001*
**Total Body Capacitance** (TBC, pF)	0.294	0.102	*0.0050*
**Age** (year)	-1.673	2.136	*0.4358*

**Figure 3:**
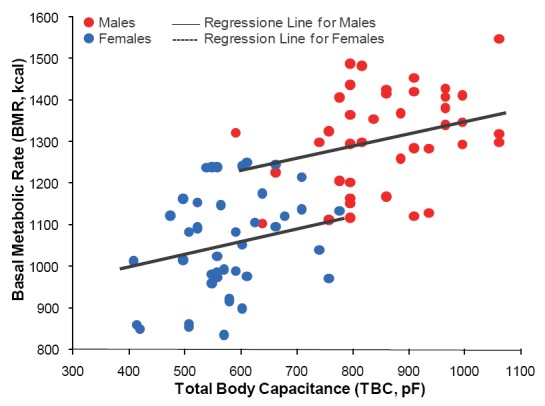
Scattergram of total body capacitance (TBC) and basal metabolic rate (BMR) for male (n=50) and for female (n = 50) participants as modeled using the multivariate regressing equation: BMR = 705.760 – 101.923 × Sex + 5.554 × Weight + 0.298 × TBC (R=0.96, SEE=48.59 kcal, and *P*<0.0001). The solid and dotted lines represent estimates of BMR computed for average body weight for males and for females (i.e., 81.06 kg and 68.94 kg, respectively).

The effect of TBC on BMR for males and for females, as modeled by Eq. 2, is shown in the scattergram of Figure 3. The solid and dotted lines represent estimations of BMR computed for average weight for males and for females (i.e., 81.06 kg and 68.94 kg, respectively). Thus, Eq. 2 correlates better than 74% with calorimetry-derived BMR (i.e., using Eq. 1), which is in line with a previous study by Cunningham (11). He found the estimated lean body mass of 233 healthy adult subjects, which is the sum of BCM and bone masses, to be the single best predictor of their BMR and to account for 70% of its variability.

**Figure 4:**
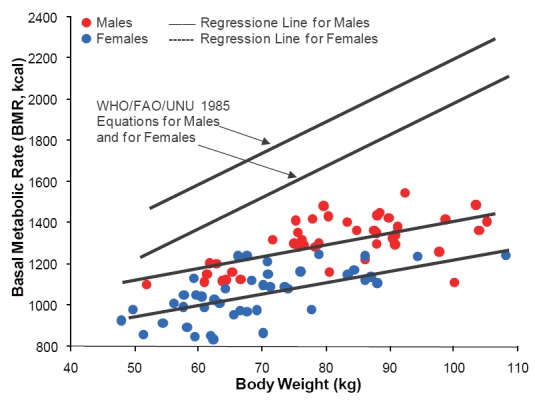
Effect of body weight on basal metabolic rate (BMR) for male (n = 50) and for female (n=50) participants as modeled using the regressing equation based on the covariates: sex, weight, and total body capacitance (TBC) (Eq. 2). The solid and dotted lines represent estimates of BMR computed for average TBC for males and for females (i.e., 856.98 pF and 577.74 pF, respectively). The solid and dotted lines at the upper part of the figure are regression plots produced using the WHO/FAO/UNU equations based only on weight for males and for females (Eq. 3 and Eq. 4) (5), which overestimated BMR values by 30% on average as compared to study regressing equation.

Figure 4 shows the effect of body weight on BMR for males and for females as modeled using Eq. 2. The solid and dotted lines represent estimations of BMR computed for average TBC (i.e., 856.98 pF for males and 577.74 pF for females, respectively) in comparison with estimations using the WHO/FAO/UNU equations for males and for females at the age range between 19 and 37 years, which are given by (5):

[Eq. 3] BMR (MJ/day)=0.0640×Weight+2.84 (Males)

[Eq. 4] BMR (MJ/day)=0.0615×Weight+2.08 (Females)

On average, Eq. 3 and Eq. 4 overestimated BMR for males and for females in comparison with calorimetry-derived BMR and BMR estimated by using Eq. 2 by more than 30%. Moreover, error analysis for simulations carried out using Eq. 3 and Eq. 4 for calorimetry-derived BMR values for males and females, showed that the SEEs were significantly higher than those produced using Eq. 2 (i.e., 159.65 kcal and 140.79 kcal versus 88.59 kcal, respectively; *P*<0.0001). That is, the error in BMR estimations using Eq. 3 and Eq. 4 were 12.2% and 11.9 for males and for females, respectively, while that for Eq. 2 was 7.49% of average calorimetry-derived BMR values. Thus, the introduction of the new parameter TBC, which can be considered as an expression for BCM, in an equation for estimating BMR improves its explanatory power and increases the accuracy of its estimations.

In conclusion, it has been shown that BCM, and not only the anthropometric parameters: age and weight, is the body compartment responsible for BMR. There are no data, in the past and present literature to the best of our knowledge, regarding the association between TBC and BMR. Therefore, in the present study we verified the existing relationship between these two parameters. A regressing equation based on the covariates: sex, weight, and TBC; was derived for estimating BMR for healthy men and women, which will have direct implications for nutritional and health status assessment on individual and population basis.
